# Biomarkers of sarcopenia in very old patients with hip fracture

**DOI:** 10.1002/jcsm.12508

**Published:** 2020-01-08

**Authors:** Carmen Sánchez‐Castellano, Sagrario Martín‐Aragón, Paloma Bermejo‐Bescós, Nieves Vaquero‐Pinto, Carmen Miret‐Corchado, Ana Merello de Miguel, Alfonso José Cruz‐Jentoft

**Affiliations:** ^1^ H. U. Ramón y Cajal IRYCIS Madrid Spain; ^2^ Departamento de Farmacología. Facultad de Farmacia. Universidad Complutense de Madrid.Departamento de Farmacología Universidad Complutense de Madrid Spain; ^3^ Hospital Fundación Jiménez Díaz Madrid Spain

**Keywords:** Hip fracture, Sarcopenia, Biomarkers, TNF‐α, Catalase activity, 20S proteasome, BuChE activity, C‐terminal agrin fragment (CAF)

## Abstract

**Background:**

Hip fracture is both a cause and a consequence of sarcopenia. Older persons with sarcopenia have an increased risk of falling, and the prevalence of sarcopenia may be increased in those who suffer a hip fracture. The aim of this study was to explore potential biomarkers (neuromuscular and peripheral pro‐inflammatory and oxidative stress markers) that may be associated with sarcopenia in very old persons with hip fracture.

**Methods:**

We recruited 150 consecutive patients ≥80 years old admitted to an orthogeriatric unit for an osteoporotic hip fracture. Muscle mass was assessed pre‐operatively using bioelectrical impedance analysis; Janssen's (J) and Masanés' (M) reference cut‐off points were used to define low muscle mass. Muscle strength was assessed with handgrip strength (Jamar's dynamometer). Sarcopenia was defined by having both low muscle mass and strength and using the European Working Group on Sarcopenia in Older People 2 definition of probable sarcopenia (low grip strength). Peripheral markers—pro‐inflammatory and oxidative stress parameters—were determined either in the plasma or in the erythrocyte fraction obtained from peripheral whole blood of every patient pre‐operatively.

**Results:**

Mean age was 87.6 ± 4.9 years, and 78.7% were women. The prevalence of sarcopenia was 11.5% with Janssen's, 34.9% with Masanés' cut‐offs, and 93.3% with the European Working Group on Sarcopenia in Older People 2 definition of probable sarcopenia. Among the four pro‐inflammatory cytokines tested in plasma, only tumour necrosis factor‐α was different (lower) in sarcopenic than in non‐sarcopenic participants using both muscle mass cut‐offs (J 7.9 ± 6.2 vs. 8.3 ± 5.8, M 6.8 ± 4.7 vs. 9.1 ± 6.2). Erythrocyte glutathione system showed a non‐significant tendency to lower glutathione levels and glutathione/oxidized glutathione ratios in sarcopenic participants compared with non‐sarcopenic subjects. Catalase activity was also lower in sarcopenic participants (J 2904 ± 1429 vs. 3329 ± 1483, M 3037 ± 1430 vs. 3431 ± 1498). No significant differences were found between groups in chymotrypsin‐like activity of the 20S proteasome, superoxide dismutase, glutathione peroxidase and butyrylcholinesterase activity, C‐terminal agrin fragment, interferon‐γ, or interleukin‐1β.

**Conclusions:**

The prevalence of sarcopenia in patients with hip fracture varies according to the definition and the muscle mass reference cut‐off points used. We did not find differences in most neuromuscular, pro‐inflammatory, or oxidative stress markers, except for lower peripheral tumour necrosis factor‐α levels and catalase activity in sarcopenic participants, which may be markers of an early inflammatory reaction that is hampered in sarcopenic patients.

## Introduction

Sarcopenia and hip fractures are both common conditions in older patients. Sarcopenia is a risk factor for falls^1^, fractures,^2^ and hip fractures.^3^ Osteoporosis and sarcopenia frequently coexist in hip fracture patients, and those with sarcopenia have an increased risk of poor outcomes, including impaired functional recovery, nursing home admission, and death.^4^, ^5^, ^6^, ^7^ A hip fracture leads to a period of immobility, which would in turn result in a loss of muscle mass.

The prevalence of sarcopenia in older patients with hip fracture ranges from 21% to 74% in men and 12% to 68% in women,^6^, ^8^, ^9^, ^10^, ^11^ depending on the definition of sarcopenia used. It is usually higher in men and in rehabilitation settings (compared with acute care).

As sarcopenia has a complex multifactorial pathogenesis involving not only age‐related changes in neuromuscular function, muscle protein turnover, and hormone levels and sensitivity but also a chronic pro‐inflammatory state and oxidative stress, studies on peripheral pro‐inflammatory and oxidative stress markers subjects might allow to identify patients at especially high risk for adverse health outcomes. Evidence on peripheral blood markers that may serve as biomarkers of sarcopenia is still sparse.^12^ It has been proposed that there will not be only one ideal biomarker, but it should be our goal to have a panel of complementary biomarkers (imaging, serum biomarkers, and functional tests) that together constitute the ideal panel of markers for the diagnosis of sarcopenia.^13^, ^14^ Some biomarkers have been described in community‐dwelling older people, as tumour necrosis factor‐α (TNF‐α), interleukin‐6 (IL‐6), and other inflammatory markers,^15^, ^16^ but these have not been explored in patients with sarcopenia and hip fracture. Only C‐terminal fragment of agrin (CAF) has been shown to be increased in hip fracture patients when sarcopenia is present.^17^

The objective of this study was to explore a range of neuromuscular and peripheral blood pro‐inflammatory and oxidative stress markers in very old patients hospitalized for the surgical management of a hip fracture and the differences between sarcopenic and non‐sarcopenic participants.

## Materials and Methods

### Participants

We prospectively recruited 150 older patients ≥80 years old who were hospitalized in a Spanish acute orthogeriatric ward for the surgical treatment of a fragility hip fracture who agreed to participate in the study. Exclusion criteria were (i) emergency surgery before informed consent could be collected, (ii) pacemaker carriers, (iii) patients with active cancer, (iv) patients who had received blood transfusion before a blood sample could be obtained, and (v) those who were unable to understand instructions to measure handgrip strength.

### Data collection

Assessment of participants included socio‐demographic data, cognitive status (Pfeiffer's questionnaire,^18^ Global Deterioration Scale—Reisberg^19^), pre‐fracture functional status (Barthel index 20 and Functional Ambulatory Categories 21), pre‐fracture nutritional status [Mininutritional Assessment—Short Form^22^; body mass index (BMI), using the last reported weight; and the Height Heel‐Knee measure technique, to avoid mobilization with the fracture], and number of reported falls and medications. This information was obtained at hospital admission as part of the routine comprehensive geriatric assessment by a geriatric nurse.

### Assessment of sarcopenia

Diagnosis of sarcopenia was based on the European Working Group on Sarcopenia in Older People (EWGSOP) criteria,^23^ adapted to this setting, where physical performance (gait speed or the Short Physical Performance Battery) could not be measured, being participants unable to walk. Muscle mass was assessed pre‐operatively using bioelectrical impedance analysis (BIA, Quantum/S Bioelectrical Body Composition Analyzer, Akern®). Skeletal muscle index (kg/m^2^) was calculated dividing absolute muscle mass by squared height. Two different cut‐off points were used to define low muscle mass, proposed by Janssen^24^ and Masanés^25^ (these are based on a Spanish reference population). Muscle strength was assessed with handgrip strength using a handheld dynamometer (Jamar®). The maximal value of three consecutive measurements in the dominant arm was used for the analysis. Low grip strength was defined as values <30 kg and <20 kg for men and women, respectively.^23^ The updated EWGSOP2 criteria were published after the study was started,^26^ emphasizing the role of muscle strength, so we decided to add the EWGSOP2 definition of probable sarcopenia, with the use of the new proposed cut‐offs for handgrip strength (<27 kg for men and <16 kg for women) and no muscle mass measure in a new *post hoc* analysis.

### Blood sampling and biochemical determinations

In each patient, fasting venous blood samples were collected in the morning, usually within 24 h after arrival. Samples were left at room temperature for 10 min and then centrifuged at 800 *g* for 10 min at 4°C. The erythrocyte fraction at the bottom of the centrifuge tube was separated from the supernatant, and the former was aliquoted and stored a −80°C until analysis. The supernatant, corresponding to the plasma fraction, was subsequently centrifuged at 3600 *g* for 20 min at 4°C in order to obtain a platelet poor plasma as the supernatant that was likewise aliquoted and stored at −80°C until analysis.

Peripheral markers (pro‐inflammatory and oxidative stress parameters) were determined either in the plasma or in the erythrocyte fraction obtained from peripheral whole blood of every patient.

#### Interferon‐γ, interleukin‐1β, interleukin‐6, and tumour necrosis factor‐α cytokines

Steady state levels of the circulating cytokines from plasma, interferon‐γ, IL‐1β, IL‐6, and TNF‐α were quantitatively determined using the Milliplex™ Multi‐Analyte Profiling Human Cytokine/Chemokine Kit for 96‐well assay (Millipore Corp, St. Louis, MO) run on a Luminex platform.^27^ For quality assurance, each sample was run twice, and the mean derived for each sample was used as the index value. Additionally, two kit‐supplied quality controls were run on each plate in duplicate and confirmed to fall within the expected range. Cytokine concentrations were calculated by reference to an eight‐point five‐parameter logistic standard curve for each cytokine.

#### Chymotrypsin‐like activity of the 20S proteasome

20S proteasomal activity was quantified in plasma fraction by monitoring the accumulation of the fluorescent cleavage product 7‐amino‐4‐methylcoumarin (AMC) from a synthetic proteasomal substrate.^28^ The fluoropeptide Suc‐Leu‐Leu‐Val‐Tyr‐AMC (Sigma‐Aldrich) was used to measure the chymotrypsin‐like activity of the proteasome.

#### Glutathione and oxidized form

As an index of redox state, levels of reduced glutathione (GSH) and oxidized form (GSSG) were determined, and the glutathione redox ratio GSH/GSSG was then calculated. GSH and GSSG were measured spectrofluorometrically in the erythrocyte fraction by using the *o*‐phthalaldehyde method, described by Senft *et al*.^29^ The concentration of GSH and GSSG in each sample was interpolated from known GSH or GSSG standards. Values were expressed as nanomole per milligram of protein.

#### Endogenous antioxidant enzymes

Endogenous antioxidant enzyme activities were measured as follows. Superoxide dismutase (SOD) was assayed by quantifying the inhibition of pyrogallol autoxidation at 420 nm.^30^ Catalase activity (CAT) was assayed in Triton X‐100 (1%)‐treated supernatants following the disappearance of H_2_O_2_ at 240 nm.^31^ SOD and CAT activities were expressed as International Units (IU) per milligram protein. One IU of SOD refers to nanogram of enzyme that produces 50% inhibition in pyrogallol autoxidation. One IU of CAT is defined as the amount of enzyme that transforms 1 μmol of H_2_O_2_ per minute at 25°C and pH 7.4. Total glutathione peroxidase (GPx) was determined following NADPH oxidation at 340 nm in the presence of excess GR, GSH, and cumene hydroperoxide as substrate.^32^ GPx activity was expressed as substrate (nmol NADPH) transformed·per minute·(per mg protein).

#### Butyrylcholinesterase activity

Assessment of butyrylcholinesterase (BuChE) activity was carried out using the Ellman's reaction,^33^ adapted for use with microtiter plates, by which the BuChE present in the plasma sample catalyzes the hydrolysis of butyrylthiocholine iodide and the resulting thiocholine product reacts with 5,5′‐dithiobis(2‐nitro‐benzoic acid) (Sigma‐Aldrich) to form a coloured anion, 5‐thio‐2‐nitro‐benzoic acid.

#### C‐terminal agrin fragment

C‐terminal agrin fragment^17^ was determined in plasma fraction using a commercially available enzyme‐linked immunosorbent assay kit (NT total CAF ELISA kit, Neurotune AG, Schlieren—Zurich, Switzerland) following the manufacturer's directions.

### Statistical analysis

A sample size of 146 participants was calculated on the basis of a sensitivity of 95% for BIA/dynamometer, a 95% confidence interval, and an expected prevalence of sarcopenia in this population of 50%.

All analyses were performed using stata software version 13.0 (Stata Inc, College Station, TX). Results for quantitative variables were described by mean and standard deviation and for qualitative variables by the absolute and relative frequency.

Bivariate analysis was used to compare the results from participants with and without sarcopenia, using the three definitions. The Student's *t*‐test or the Mann–Whitney *U* test was applied for quantitative variables, and the *χ* ^2^ test was used for qualitative variables. We measured concordance between the methods to detect sarcopenia using Cohen's kappa coefficient.

### Ethics

The study was approved by the Ethics Committee of our hospital. All participants signed a written consent before enrolment. In patients with cognitive impairment, a proxy family member signed the consent following local regulations.

## Results

### Characteristics of participants and prevalence of sarcopenia

Baseline characteristics of the 150 participants are listed in *Table* 1, and we previously published these results in detail elsewhere.^34^ In brief, mean age was 87.6 ± 4.9 years, and 78.7% were women. The prevalence of sarcopenia was 11.5% using Janssen's cut‐off points and 34.9% using Masanés' cut‐off points within the EWGSOP 2010 definition. Characteristics of sarcopenic and non‐sarcopenic participants using both definitions are also listed in *Table* 1. The concordance between both measures of sarcopenia prevalence (based on Janssen's and Masanés' cut‐off points, respectively) was weak, with a kappa coefficient 0.316. Sarcopenic and non‐sarcopenic participants were similar in most baseline characteristics (the only exception was a reduced BMI in sarcopenic participants with sarcopenia defined by Masanés' cut‐offs). The prevalence of probable sarcopenia using the EWGSOP2 definition was 93.3% (94% in women and 90.3% in men). Comparing both groups with this criterion, those with probable sarcopenia had more probability of being woman, suffering dementia (Global Deterioration Scale scale), suffering a perthrocanteric fracture, and having a worse functional situation and previous mobility, measured with Barthel and Functional Ambulatory Categories scales, respectively.

**Table 1:** Baseline characteristics of participants

	**All participants**	**Sarcopenia (Janssen)**		**Sarcopenia (Masanés)**		**Sarcopenia (low handgrip EWGSOP2)**	
		Yes	No	Yes	No	Yes	No
**N**	150	17 (11.5%)	133 (88.5%)	52 (34.9%)	98 (65.1%)	140 (93.3%)	9 (6.7%)
**Age** (years), mean ± SD	87.6 ± 4.9	87.4 ± 5.3	87.7 ± 4.9	87.7 ± 4.9	87.8 ± 5.0	87.8 ± 4.8	86.2 ± 6
**Gender** (% women)	78.7%	70.6%	80.2%	82.4%	76.8%	**79.9%**	**70%**
**Type of fracture**							
Perthrocanteric	53.0%	29.4%	55.4%	44.0%	56.8%	**51.4%**	**70%**
Subcapital	37.6%	58.8%	35.4%	42.0%	35.8%	**38.4%**	**30%**
Subthrocanteric	9.4%	11.8%	9.2%	14.0%	7.4%	**10.2%**	**0%**
**Handgrip strength**							
Mean	8.1 ± 6.6	11.9 ± 8.4	7.6 ± 6.3	8 ± 6.4	8.1 ± 6.8	**7.1 ± 5.5**	**21.2 ± 7.2**
Women	6.6 ± 5	8.5 ± 6.3	6.4 ± 4.8	6.4 ± 5.1	6.7 ± 5.1	**6 ± 4.4**	**16.9 ± 6.7**
Men	13.6 ± 9	20 ± 7.2	12.4 ± 8.8	15.3 ± 7.3	12.9 ± 9.6	**11.7 ± 7.1**	**31.3 ± 3.1**
**MNA®‐SF**							
Malnutrition	12.6%	11.8%	13%	9.8%	13.7%	13.7%	0%
Risk of malnutrition	49.0%	58.8%	47.3%	60.8%	43.2%	49.6%	40%
Normal	38.4%	29.4%	39.7%	29.4%	43.2%	36.7%	60%
**BMI**							
<18.5	22.9%	33.3%	21.7%	36.0%	16.0%	23.9%	10%
18.5‐24.9	35.4%	40.0%	34.9%	42.0%	31.9%	35%	40%
25‐29.9	26.4%	20.0%	27.1%	14.0%	33.0%	26.1%	30%
≥ 30	15.3%	6.7%	16.3%	8.0%	19.1%	15%	20%
**Gait (FAC)**							
Independent (4‐5)	77.5%	88.2%	77.1%	78.4%	77.9%	**76.3%**	**100%**
Dependent (0‐3)	22.5%	11.8%	22.9%	21.6%	22.1%	**23.7%**	**0%**
**BADL (Barthel)**							
Independent (100)	14.2%	23.5%	13.2%	20.0%	11.7%	**11.6%**	**50%**
Mild dependence (60‐95)	66.9%	64.7%	67.4%	66.0%	68.1%	**67.4%**	**50%**
Moderate dependence (40‐55)	13.5%	5.9%	14.0%	6.0%	16.0%	**14.5%**	**0%**
Severe dependence (0‐35)	5.4%	5.9%	5.5%	8.0%	4.3%	**5%**	**0%**
**Cognition (Pfeiffer)**							
Normal	53.7%	50.0%	53.3%	54.8%	52%	50.5%	88.9%
≥ 3 errors	46.3%	50.0%	46.7%	45.2%	48%	49.5%	11.1%
**Reisberg GDS**							
No dementia	62.1%	50.0%	63.2%	60.0%	63.3%	**60.2%**	**85.7%**
Mild dementia	21.6%	37.5%	19.8%	25.7%	19.0%	**23.1%**	**0%**
Moderate‐severe dementia	16.4%	12.5%	17.0%	14.3%	17.7%	**16.7%**	**14.3%**
**Prescribed drugs** (n)	7.2 ± 3.6	7.7 ± 2.6	7.2 ± 3.8	7.7 ± 3.6	7.0 ± 3.7	7.3 ± 3.7	6.8 ± 2.8
**Previous falls** ((≥3)	40.0%	1.4 ± 2.0	1.9 ± 2.8	2.0 ± 2.6	1.9 ± 2.8	1.9 ± 2.6	1.7 ± 4.5

SD = standard deviation. MNA‐SF = mini nutritional assessment‐short form. BMI = body mass index. FAC = functional ambulation categories. BADL = basic activities of daily living. GDS= global deterioration scale.

### Peripheral markers

**Figure 1:**
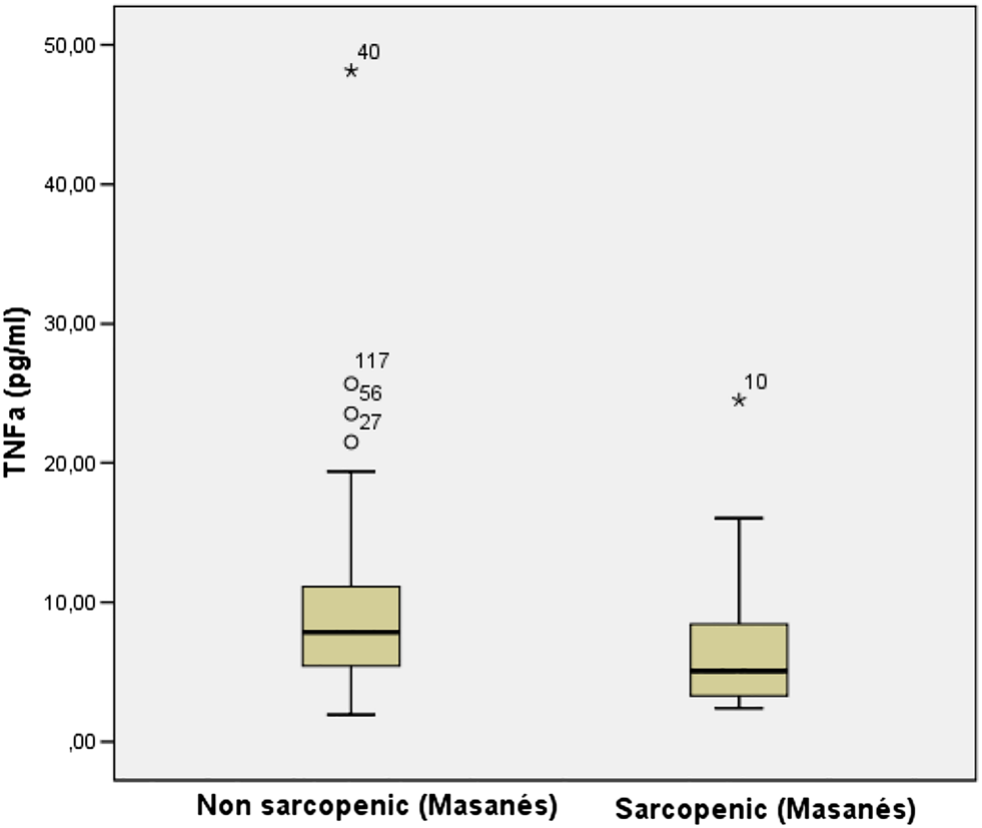
TNF‐α levels in sarcopenic and non‐sarcopenic participants

**Figure 2:**
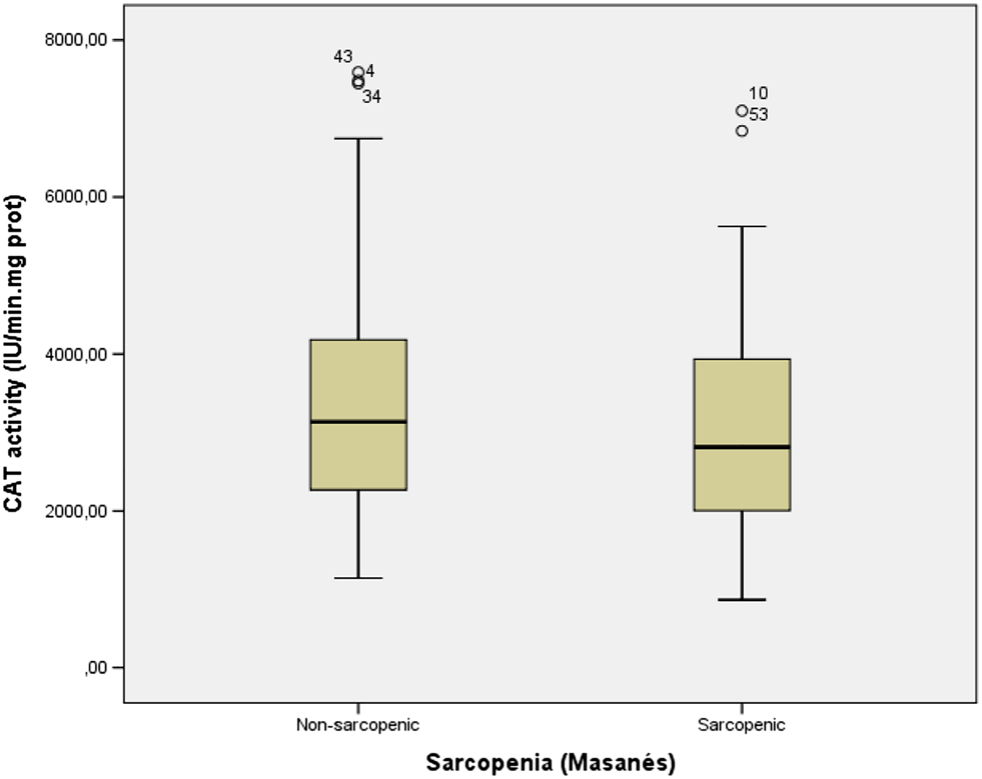
CAT activity levels in sarcopenic and non‐sarcopenic participants

Among the four pro‐inflammatory cytokines tested (interferon‐γ, IL‐1β, IL‐6, and TNF‐α) in plasma samples, only TNF‐α was significantly different in sarcopenic and non‐sarcopenic participants (7.9 ± 6.2 vs. 8.3 ± 5.8 with Janssen's cut‐offs; 6.8 ± 4.7 vs. 9.1 ± 6.2, Masanés' cut‐offs) with muscle mass measures, but not using the EWGSOP2 definition (*Table* 2 and *Figures* 1 and 2).

**Table 2:** Peripheral markers in sarcopenic vs. non‐sarcopenic participants

Biomarker	EWGSOP Sarcopenia (Janssen)	EWGSOP No sarcopenia (Janssen)	EWGSOP Sarcopenia (Masanés)	EWGSOP No sarcopenia (Masanés)	EWGSOP2 probable sarcopenia	EWGSOP2 no sarcopenia
IFNγ (pg/ml)	7.8 ± 6.7	10.0 ± 14.8	9.9 ± 21.4	9.8 ± 8.8	9.9 ± 14.5	6.9 ± 5.7
IL‐1β (pg/ml)	1.0 ± 0.7	1.4 ± 1.5	1.0 ± 0.8	1.5 ± 1.7	1.4 ± 1.5	0.8 ± 0.6
IL‐6 (pg/ml)	15.3 ± 12.7	12.2 ± 10.3	12.3 ± 12.9	12.8 ± 9.3	12.6 ± 10.9	10.8 ± 5.5
TNFα (pg/ml)	**7.9 ± 6.2**	**8.3 ± 5.8**	**6.8 ± 4.7**	**9.1 ± 6.2**	**8.2 ± 5.9**	**8.7 ± 4.4**
20S (nmol AMC/mg protein)	13.1 ± 3.7	13.3 ± 5.0	12.9 ± 4.0	13.6 ± 5.2	13.2 ± 4.9	14.2 ± 4.9
nmol GSH/mg protein	48.4 ± 34.4	53.0 ± 25.4	51.8 ± 30.1	53.4 ± 24.5	53.1 ± 26.7	44 ± 21
nmoles GSSG/mg protein	2.1 ± 0.9	2.6 ± 1.5	2.6 ± 1.3	2.6 ± 1.6	2.6 ± 1.5	1.9 ± 1.1
GSH/GSSG	22.8 ± 9.3	25.7 ± 17.8	22.5 ± 12.3	27.0 ± 19.1	25 ± 16.9	29.3 ± 19.2
CAT activity (IU/min.mg protein)	**2904.1 ± 1428.7**	**3328.5 ± 1482**	**3037.2 ± 1429.6**	**3431 ± 1498**	**3267.3 ± 1500.9**	**3433.8 ± 1045.6**
SOD activity (U/mg protein)	14.7 ± 8.5	15.1 ± 10.8	13.8 ± 9.6	15.6 ± 11.0	14.8 ± 10.1	20.6 ± 15.5
GPx activity (nM NADPH/min.mg protein)	82.3 ± 37.5	104.5 ± 81.9	97.5 ± 81.4	104.8 ± 77.6	102.1 ± 79.3	107 ± 65.5
BuChE activity (U/mg protein)	2.1 ± 0.9	2.4 ± 1.0	2.5 ± 0.9	2.4 ± 1.0	2.4 ±0.9	2.7 ±1.7
CAF (pM)	536.1 ± 1054.1	440.6 ± 632.4	439.2 ± 681.2	493.0 ± 768.2	456.4 ± 727.3	378.7 ± 229.2

IFNγ: interferon gamma. IL: interleukin. TNFα: tumor necrosis factor alpha. 20S: 20S proteasome. GSH: glutathione reduced form. GSSG: glutathione oxidized form. GSH/GSSG: glutathione redox ratio. CAT: catalase. SOD: superoxide dismutase. GPx: glutathione peroxidase. NADPH: Nicotinamide adenine dinucleotide phosphate reduced. BuChE: butyrylcholinesterase. CAF: C‐terminal agrin fragment. EWGSOP: European working group on sarcopenia in older people. In bold: significant results (p < 0.05).

As for the plasma chymotrypsin‐like activity of the 20S proteasome, no significant differences were achieved between sarcopenic and non‐sarcopenic individuals, determined upon either of the cut‐offs.

Analyses of the erythrocyte glutathione system showed lower GSH levels and GSH/GSSG ratios in sarcopenic patients compared with non‐sarcopenic subjects, though differences between both groups were not significant.

Amidst the peripheral endogenous antioxidant defenses determined in the erythrocyte fraction, CAT was significantly lower in sarcopenic versus non‐sarcopenic participants (2904.1 ± 1428.7 vs. 3328.5 ± 1482.0, Janssen's cut‐offs; 3037.2 ± 1429.6 vs. 3431 ± 1498, Masanés' cut‐offs), again not with the EWGSOP2 definition (Table 2). All subjects had CAF values above the normal CAF upper cut‐off value (>181.36 pM for men and >139.1 pM for women, Hettwer *et al*., 2013).

With regard to the plasma BuChE activity, significant differences were not found between sarcopenic and non‐sarcopenic participants upon either of the cut‐offs.

No significant differences were found in any of the biomarkers between participants with and without probable sarcopenia using the EWGSOP2 definition.

## Discussion

We have explored a large set of biomarkers in patients with a recent hip fracture with and without sarcopenia and shown that only TNF‐α and CAT levels were different between groups. The rest of the neuromuscular and peripheral pro‐inflammatory and oxidative stress markers explored did not differ.

Impaired immune response is a frequent finding in elderly people, and a relationship between age and some immune impairments in patients with hip fracture has been observed.^35^ For instance, increased serum IL‐6 levels are positively associated with disability rate and mortality^36^ and negatively correlated to the recoveries of walking and muscle function in the fracture healing process in elderly women.^37^ Inflammatory reaction plays an important role in the development of elderly sarcopenia; particularly, plasma levels of the pleiotropic cytokine TNF‐α have been found to be significantly higher in sarcopenia subjects from a cross‐sectional study that included 441 subjects over 60 years^15^; IL‐6 levels were also different in this study. Another study found that elevated levels of TNF‐α were associated with a decline in muscle mass and strength.^38^ TNF‐α is a pro‐inflammatory cytokine with short length that acts directly and indirectly on muscle degradation process, induces the production of other catabolic cytokines triggering a second inflammatory cycle.^16^

At the same time, however, there is increasing evidence that inflammation plays a vital role in early fracture repair.^39^ Intracellular and extracellular regulators of healing mechanisms, such as cytokines, have been described to possess significant roles in facilitating optimal recovery^40^ in murine models; TNF‐α, IL‐1β, and IL‐6 are expressed at the fracture site within 24 h of injury. Overall, cytokines are most essential in the intermediate stages (6–24 h), while increased intracellular protein expression and phosphorylation are more prominent later when new tissue formation is essential**.** The influence of TNF‐α on fracture healing *in vivo* has been investigated using a murine model.^38^ Resident stromal cells present in muscle seem to be an important source of osteoprogenitor cells. Uniquely, it has been demonstrated that pro‐inflammatory cytokines, in particular TNF‐α, are crucial in fracture healing. This takes place in a concentration‐dependent manner. *In vivo*, there is likely to be a concentration gradient of TNF‐α, with progressive reduction radially from the fracture site. In our study, we found significant higher plasma levels of TNF‐α in non‐sarcopenic versus sarcopenic participants. This finding may explain the slower recovery of sarcopenic patients with hip fracture: following cell migration to the fracture site, TNF‐α, at a higher concentration, may then inhibit further cell migration and promote local osteogenesis.

As a long‐term muscle protein unbalance between the rate of protein synthesis and the rate of its breakdown might sustain age‐related loss of muscle tissue, we assessed the status of the protein degradation machinery in skeletal muscle of our patients. The ubiquitin–proteasome pathway is the most important mechanism for regulation of protein quality control in skeletal muscle cells.^41^ Proteins are targeted by an enzyme system that binds them to a polypeptide ubiquitin. The ubiquitinized proteins are then transferred to the proteasome complex and degraded into short peptides that are finally recycled as free intracellular amino acids. Intriguingly, this pathway is promoted by inflammatory cytokines, such as TNF‐α and IL‐6, as well as by reactive oxygen species (ROS), among others.^42^ Having found higher TNF‐α levels in the non‐sarcopenic participants, we wondered whether this group had likewise achieved higher proteasome activation. The most widely known function of the proteasome is protein degradation through the 26S ubiquitin–proteasome system, responsible for the vast majority of protein degradation during homeostasis. However, the proteasome also plays an important role in adaptive immune responses and adaptation to oxidative stress. The unbound 20S proteasome, the core common to all proteasome conformations, is the main protease responsible for degrading oxidized proteins.^43^ The chymotrypsin‐like activity of the 20S proteasome was evaluated in our participants, and against our expectations, no differences in 20S activity between groups were observed.

A progressive increase in cellular oxidative stress during aging has been implicated as a major contributor to sarcopenia. Accordingly, a direct correlation between muscle ROS and the muscle mass during normal aging and in muscle diseases has been reported.^44^ In addition, antioxidant intake and circulating levels as well as markers of oxidative damage have been variously correlated with sarcopenia,^45^ bone remodelling processes, fractures, and physical function in older adults.^46^, ^47^, ^48^ We found that one of the endogenous defenses to oppose increased stress in older muscle cells, CAT, may be altered in sarcopenic compared with non‐sarcopenic participants. This finding is in agreement with Sullivan‐Gunn and Lewandowski^49^ who found that elevated hydrogen peroxide and decreased CAT and GPx are associated with aging sarcopenia. They also suggested that hydrogen peroxide is the key ROS in the onset of sarcopenia and that the decline in antioxidant protection by CAT (and GPx) is indicative of antioxidant dysfunction and may be a major contributing factor in the development or onset of sarcopenia. The presence of sarcopenia in our hip fractured patients is possibly associated with a less vigorous antioxidant defense response; an increased in misfolded protein stress in sarcopenic participants would be expected in comparison with non‐sarcopenic participants, although no differences in 20S proteasome activity were observed. Reduced cellular defense markers and increased level of misfolded proteins in sarcopenic muscle might contribute to intracellular accumulation of ROS.

Butyrylcholinesterase is an α‐glycoprotein found in the central and peripheral nervous system, in most tissues and liver, relevant in some physiological and pathological conditions,^50^ particularly in regulating the immune response during inflammation.^51^ Low plasma BuChE level has been found in protein–energy malnutrition, during stress and (chronic and acute) inflammation, and as a result, it has been claimed to be included in routine clinical diagnostic procedures to evaluate patient clinical conditions such as inflammation and/or protein–energy malnutrition.^52^ Interestingly, peripheral levels of BuChE have been found to be related to muscle mass and strength in older subjects and, furthermore, lower in sarcopenic versus non‐sarcopenic elderly subjects.^53^ We did not confirm this finding in very old persons with hip fracture.

During neuromuscular remodelling, agrin, a large proteoglycan in the synaptic cleft component of the neuromuscular junction, is split by the neurotrypsin enzyme into a 22 kDa agrin soluble fragment that contains the CAF. The degree of release of CAF into the blood is proportional to the increase in neurotrypsin activity and the reduction in neuromuscular junction strength.^51^ In older adults with sarcopenia, serum concentrations of CAF have been shown to be elevated compared with adult controls of the same age.^54^, ^55^ Because this process seems to be triggered by cellular damage mediated by oxidative stress,^56^ we expected a higher peripheral CAF concentration in patients with a lower endogenous antioxidant capacity, that is to say, in sarcopenic subjects. First, in our study, the entire sample (sarcopenic and non‐sarcopenic) presented elevated CAF levels, above normal values,^55^ indicating signs of neuromuscular junction degeneration. Second, regarding the presence of sarcopenia, peripheral levels of CAF appeared to be higher in hip fractured sarcopenic patients compared with hip fractured non‐sarcopenic patients upon Janssen's cut‐offs. Although no significant differences were achieved, our CAF level pattern is somehow in concordance with data reported by Marzetti *et al*., 17 who observed higher levels in hip fractured patients with sarcopenia versus the equivalent non‐sarcopenic group.

Hip fracture is an acute, fluctuating condition. Acute and chronic conditions may have a role in the fall that leads to the fracture, and immediate adaptive physiological changes are expected to happen after the fracture. This is compounded with immobility, hospital admission, pain control, treatment of co‐morbid conditions, and surgical procedures. Most of these can have an impact on the levels of biomarkers, which are not expected to remain stable along the time after the hip fracture happens, so interpretation of our findings is complex. Our intention was to measure biomarkers as early as possible to reduce the number of confounders and reflect the hip fracture acute inflammatory changes, which may be associated with mortality.^57^ If a predictive value of pre‐operative biochemical parameters is confirmed, they may offer an opportunity for both risk stratification and individualized therapeutic decisions.^58^

We acknowledge that this study had some limitations. Very old age may attenuate differences between groups, as aging is associated with many of the proposed mechanisms that produce sarcopenia. The fact that all subjects had an acute severe disease that triggers its own inflammatory and repair mechanisms may act as an additional confounder. Also, hydro and electrolyte disturbances may appear after a hip fracture that may bias body composition assessment by BIA. Muscle mass measures may also have been altered in participants with heart or renal failure. Pre‐operative changes in BIA in hip fracture patients have not been well studied, but muscle mass has been found to be lower in such patients compared with healthy age, gender, and BMI matched controls.^59^ Literature suggests that reliability of BIA may be reduced in surgical patients due to hydro and electrolyte disturbances.^60^ The presence of infections, delirium, or pain and the use of medications that can affect the muscle strength may also falsely increase the number of participants with sarcopenia. Finally, we did not obtain a measure of frailty,^61^ which may have helped in the categorization of participants.

## Conclusions

Up to one third of patients with hip fracture may be sarcopenic on hospital admission, although prevalence depends heavily on the cut‐off points used to define low muscle mass. We explored a set of biomarkers and found no differences in most neuromuscular, pro‐inflammatory, or oxidative stress markers, except for a lower peripheral TNF‐α levels and CAT in sarcopenic participants, which may be markers of an early inflammatory reaction that is hampered in sarcopenic patients. These two biomarkers, together with CAF, merit further exploration in other settings (including the post‐acute phase of hip fracture) and other groups of older persons.

## Conflict of interest

All authors declare that they have no conflict of interest.
